# Prognostic value of pretest probability of heart failure with preserved ejection fraction in patients with coronary artery disease: an insight from the CLIDAS-PCI database

**DOI:** 10.1007/s12928-026-01286-y

**Published:** 2026-05-16

**Authors:** Tomoaki Nishikawa, Shunsuke Tamaki, Kazuhisa Nishimura, Yasutaka Ihara, Akinori Higaki, Hiroshi Kawakami, Katsuji Inoue, Shuntaro Ikeda, Osamu Yamaguchi, Naoyuki Akashi, Takahide Kohro, Tomoyuki Kabutoya, Kazuomi Kario, Arihiro Kiyosue, Masaharu Nakayama, Yoshihiro Miyamoto, Kenichi Tsujita, Hideo Fujita, Tetsuya Matoba, Ryozo Nagai

**Affiliations:** 1 Department of Cardiology, Pulmonology, Hypertension, and Nephrology, Ehime University Graduate School of Medicine, 454 Shitsukawa, Toon, Ehime, 791-0295 Japan; 2 Clinical Research Promotion Unit, Clinical Therapeutic Trial Center, Ehime University Hospital, Toon, Japan; 3 Division of Cardiovascular Medicine, Jichi Medical University Saitama Medical Center, Shimotsuke, Japan; 4 Department of Clinical Informatics, Jichi Medical University School of Medicine, Shimotsuke, Japan; 5 Division of Cardiovascular Medicine, Department of Medicine, Jichi Medical University School of Medicine, Shimotsuke, Japan; 6 Department of Cardiovascular Medicine, University of Tokyo Hospital, Bunkyo-ku, Japan; 7 Department of Medical Informatics, Tohoku University Graduate School of Medicine, Sendai, Japan; 8 Open Innovation Center, National Cerebral and Cardiovascular Center, Suita, Japan; 9 Department of Cardiovascular Medicine, Graduate School of Medical Sciences, Kumamoto University, Kumamoto, Japan; 10 Department of Cardiovascular Medicine, Kyushu University Graduate School of Medical Sciences, Fukuoka, Japan; 11 Jichi Medical University School of Medicine, Shimotsuke, Japan

**Keywords:** Coronary artery disease, HFpEF-ABA score, Prognosis

## Abstract

Coronary artery disease (CAD) is a major risk factor for the development of heart failure (HF) with preserved ejection fraction (HFpEF) and is associated with increased mortality. However, an optimal strategy to screen for HFpEF among patients with CAD has not yet been established. The HFpEF-ABA score was introduced to estimate the pretest probability of HFpEF and was shown to predict adverse HF events. This retrospective multicenter cohort study included patients registered in the Clinical Deep Data Accumulation System database who underwent percutaneous coronary intervention from April 2013 to March 2019. Patients with a left ventricular (LV) ejection fraction ≥ 50% and no known history of HF were included. Age, body mass index, and a history of atrial fibrillation were used to calculate the HFpEF-ABA score, and patients were dichotomized at a 50% threshold for descriptive risk stratification. The primary endpoint was a composite of all-cause death and unplanned HF hospitalization. Among 3307 patients, those with high HFpEF-ABA scores were more likely to have hypertension, renal dysfunction, anemia, larger left atrial size, higher LV mass index, and elevated brain natriuretic peptide levels, consistent with an HFpEF phenotype. Over a median follow-up of 721 days, 275 patients experienced the primary endpoint. The HFpEF-ABA score was independently associated with the primary endpoint as both a continuous and a categorical variable (hazard ratio: 1.07 [95% confidence interval: 1.00–1.14], *P* = 0.043, and 1.37 [1.07–1.76], *P* = 0.015, respectively). The HFpEF-ABA score identifies CAD patients with an HFpEF phenotype and predicts adverse outcomes.

**Graphical abstract:**

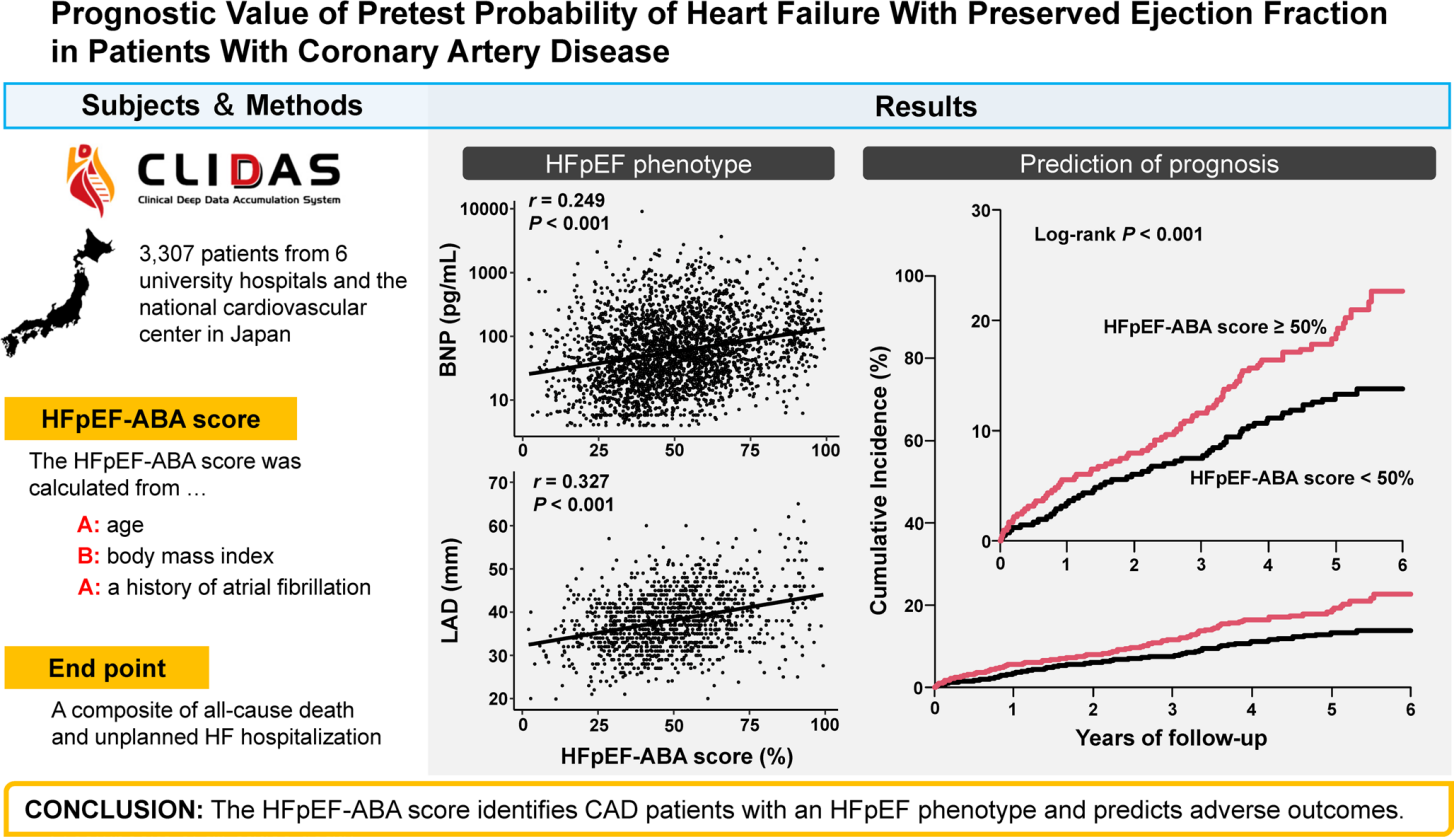

**Supplementary Information:**

The online version contains supplementary material available at 10.1007/s12928-026-01286-y.

## Introduction

Coronary artery disease (CAD) remains a major contributor to global mortality [1]. Owing to its high prevalence and associated fatal outcomes, the identification of prognostic risk factors and the implementation of appropriate interventions are essential. Recent studies have highlighted heart failure (HF) as one such prognostic factor in patients with CAD. CAD is a well-established risk factor for the development of HF [2], and once HF complicates the course of CAD, it further worsens prognosis [3–5], underscoring the synergistic interaction between the two conditions. Given their combined risk for adverse clinical outcomes, the early recognition of HF in patients with CAD may facilitate the timely initiation of appropriate therapies, ultimately leading to improved prognosis.

A substantial proportion of patients with CAD who later develop HF have preserved left ventricular ejection fraction (LVEF), referred to as HF with preserved ejection fraction (HFpEF) [2–5]. In light of the prognostic impact of HF in patients with CAD, an optimal tool to screen for preclinical or early-stage HFpEF in this population is highly warranted. Diagnostic scores and algorithms have been proposed to estimate the probability of HFpEF using combinations of echocardiographic and clinical variables [6, 7]; however, high-quality echocardiographic data are often unavailable in the routine management of CAD [8]. Therefore, a simple and readily applicable screening strategy is of clinical importance.

The recently introduced HFpEF-ABA score, derived from age, body mass index (BMI), and a history of atrial fibrillation (AF), allows easy estimation of the pretest probability of HFpEF and has also been shown to predict adverse HF outcomes [9]. In this study, we aimed to evaluate the utility of the HFpEF-ABA score for risk stratification in patients with CAD who underwent percutaneous coronary intervention (PCI), using the Clinical Deep Data Accumulation System (CLIDAS)-PCI database [10, 11].

## Methods

### Clinical deep data accumulation system

The CLIDAS database is a multimodal system that directly acquires clinical data from hospital information systems (HIS). Implemented in six university hospitals and the national cardiovascular center in Japan, CLIDAS was developed as part of the Japan Ischemic Heart Disease Multimodal Prospective Data Acquisition for Precision Treatment project launched in 2015. This project aimed to establish a standardized electronic system based on HIS to capture medical records and other clinical data for clinical studies. Briefly, data from HIS, picture archiving and communication systems, and physiology servers are linked to a multi-purpose clinical data repository system (MCDRS) through the Standardized Structured Medical Record Information eXchange2 (SS-MIX2) standard and extended storage. Data managers and researchers at each facility collect patients’ background information and follow-up data. After anonymization, the facilities send their data to the CLIDAS server through their individual MCDRS servers. CLIDAS collects data following the SS-MIX/SS-MIX2 standard format developed by Japan’s Ministry of Health, Labour and Welfare. Finally, researchers analyze the data stored on the CLIDAS server. The study protocol adhered to the ethical guidelines in the Declaration of Helsinki and was approved by the institutional ethics committees of all participating facilities. Since the CLIDAS data was anonymized, the requirement for informed consent was waived.

### Study patients and data collection

This was a retrospective, multicenter, observational cohort study. We registered patients with CAD who had undergone PCI at seven hospitals between April 2013 and March 2019. This study period was determined as the period from the time when available data could be consistently obtained from each facility to the end of the ethics committee’s accreditation. In the CLIDAS-PCI database, index PCI was defined as the first PCI procedure within the study period if multiple vessels were treated in a staged manner. We included patients with a LVEF ≥ 50% at baseline and no known history of HF. A history of HF was defined as a prior hospitalization for HF [11].

To increase the acquisition rate of laboratory and echocardiographic data, baseline laboratory values were calculated as the average of data collected from 60 days before and 30 days after the index PCI, and echocardiographic data were taken from the assessment closest to the date of the index PCI within this period [10, 11]. Acute coronary syndrome (ACS) included ST-segment elevation myocardial infarction, non-ST-segment elevation myocardial infarction, and unstable angina. Chronic coronary syndrome (CCS) was defined as cases of PCI for significantly stenosed coronary lesions not caused by ACS. Hypertension was defined as a systolic blood pressure ≥ 140 mmHg, diastolic blood pressure ≥ 90 mmHg, or a medical treatment of hypertension at index PCI. Diabetes was defined as a hemoglobin A1c level ≥ 6.5%, casual blood glucose level ≥ 200 mg/dL, fasting blood glucose level ≥ 126 mg/dL, or medical treatment for diabetes at index PCI. Dyslipidemia was defined as a medical treatment for dyslipidemia at the index PCI or description of dyslipidemia on electronic medical records. Anemia was defined as a hemoglobin level of < 13.0 g/dL in men and < 12.0 g/dL in women, according to the World Health Organization criteria [12]. LVEF was calculated using the modified Simpson’s rule; however, the Teichholz method was used for the measurement if the data of the modified Simpson’s rule were missing. The estimated glomerular filtration rate was calculated using the modified isotope dilution mass spectrometry traceable modification of diet in renal disease study equation with a Japanese coefficient [13]. For medication status, we identified the prescriptions between 10 days before and 10 days after the index PCI [11].

The HFpEF-ABA score was calculated from age, BMI, and history of AF, as previously reported [9]. Log odds of HFpEF was calculated as − 7.788751 + 0.062564 × age + 0.135149 × BMI + 2.040806 × AF, where age was expressed in years, BMI in kg/m², and AF was coded as present (= 1) or absent (= 0). Probability of HFpEF was then derived from log odds as *P* = 1 / (1 + e^(-log odds)). For descriptive risk stratification and graphical presentation, patients were additionally dichotomized using an intuitive 50% probability threshold (≥ 50% vs. < 50%) to facilitate interpretation and maintain adequate group sizes.

### Study endpoints and follow-up

The primary endpoint of this study was a composite of all-cause death and unplanned hospitalization for worsening HF. Secondary endpoints were the individual components of the primary endpoint. Follow-up duration was calculated from the date of the index PCI to the event date or the last follow-up. Patients were followed for up to 6 years. Events were confirmed at each facility through patient records, phone calls, and letters.

### Statistical analysis

The demographic and clinical characteristics of the patients were summarized using the median and interquartile range for continuous variables, and numbers (percentages) for categorical variables. The Mann-Whitney *U*-test or Kruskal-Wallis test, as appropriate, and chi-square test were used to compare differences in continuous and categorical variables, respectively. The associations of HFpEF-ABA score with B-type natriuretic peptide (BNP) levels and left atrial dimension (LAD) were evaluated by the Pearson’s correlation coefficient. The association between HFpEF-ABA score and the primary endpoint was evaluated using Cox proportional hazards regression analysis. In the primary analysis, the HFpEF-ABA score was modeled as a continuous variable (per 10% increase in predicted probability) (Model 1). In an additional analysis for descriptive stratification, the score was dichotomized at a 50% probability threshold (≥ 50% vs. < 50%) and entered as a categorical variable (Model 2). As a sensitivity analysis, the score was additionally dichotomized at a 75% probability threshold (≥ 75% vs. < 75%), which has been used in a recent study [14]. A multivariable Cox model for the endpoints was adjusted for 19 baseline characteristics: sex, ACS, hypertension, diabetes mellitus, dyslipidemia, stroke, peripheral arterial disease, cancer, current tobacco use, previous myocardial infarction, heart rate, left ventricular (LV) end-diastolic dimension, LVEF, LV mass index (LVMI), white blood cell count, hemoglobin, blood urea nitrogen, estimated glomerular filtration rate, and BNP; these variables were considered clinically important or previously shown to have prognostic significance [15–18]. Age, BMI, and AF were not included in the multivariable Cox model because they were used for the calculation of HFpEF-ABA score. Multiple imputation using predictive mean matching was employed to address missing values of the variables included in the Cox model [19]. Additional Cox analyses included subgroup analyses according to clinical presentation (ACS vs. CCS) with formal interaction testing and sensitivity analyses using covariates with *P* < 0.05 in univariable analysis. Exploratory analyses also included a Cox model restricted to the low-score subgroup and a component-level Cox model. Discrimination analyses for the primary endpoint were performed using Harrell’s C statistics for time-to-event outcomes derived from Cox models. These analyses evaluated a forced-in baseline clinical model before and after addition of the HFpEF-ABA score, with likelihood ratio testing for nested model comparison; HFpEF-ABA, LVMI, and BNP as single markers and models combining HFpEF-ABA with LVMI or BNP; and AF alone versus HFpEF-ABA alone. The cumulative incidence of the primary endpoint or all-cause death was estimated using the Kaplan–Meier method and compared between groups with the log-rank test. The cumulative incidence of unplanned hospitalization for worsening HF was estimated using the Fine–Gray method to account for mortality as a competing risk. BNP was logarithmically transformed for the correlation analysis, and both blood urea nitrogen and BNP were logarithmically transformed for the Cox analyses. All analyses were performed using R version 4.4.2 (R Foundation for Statistical Computing, Vienna, Austria). All reported *P* values are two-tailed and were considered statistically significant at < 0.05.

## Results

**Fig. 1:**
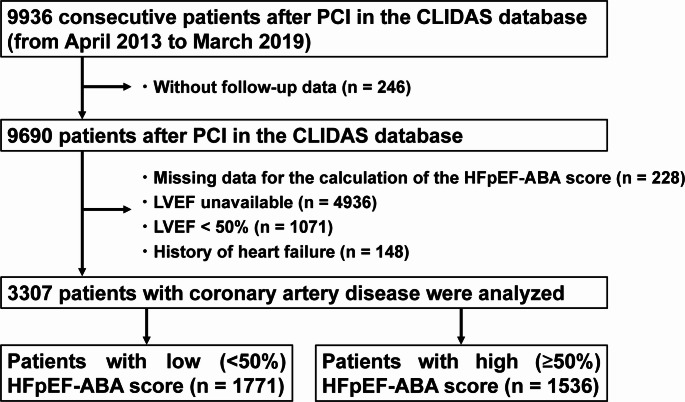
Study flow diagram. Flow diagram of the study population. LVEF, left ventricular ejection fraction; PCI, percutaneous coronary intervention

Of the 9936 consecutive patients in the CLIDAS-PCI database, those with missing follow-up data (*n* = 246), missing data for the calculation of the HFpEF-ABA score (*n* = 228), LVEF < 50% (*n* = 1071), unavailable LVEF data (*n* = 4936), or a history of HF (*n* = 148) were excluded. Consequently, a total of 3307 patients were included in the present analysis. Among them, 1771 patients had a low HFpEF-ABA score, and 1536 had a high HFpEF-ABA score (Fig. 1).

### Baseline characteristics

The baseline characteristics of the 3307 patients are summarized in Table 1. Patients with a high HFpEF-ABA score were older and more likely to be female, had a higher BMI, and had a higher prevalence of hypertension, AF, chronic kidney disease, stroke, anemia, cancer, past PCI, and past coronary artery bypass grafting, but a lower prevalence of ACS, hemodialysis, and current tobacco use. Additionally, LAD, LVMI, interventricular wall thickness at end-diastole, left ventricular posterior wall thickness at end-diastole were greater, and glycated hemoglobin, creatinine, blood urea nitrogen, and BNP levels were higher, whereas diastolic blood pressure, heart rate, white blood cell count, hemoglobin, albumin, total cholesterol, low-density lipoprotein cholesterol, high-density lipoprotein cholesterol, and estimated glomerular filtration rate were lower in patients with a high HFpEF-ABA score. Patients with a high HFpEF-ABA score also had a higher prescription rate of diuretic. There were no significant differences in the other baseline characteristics.

**Table 1:** Baseline Characteristics

Characteristics	Total (*n* = 3307)	Missing n (%)	HFpEF-ABA Score		*P* value
			Low (< 50%) (*n* = 1771)	High (≥ 50%) (*n* = 1536)	
Age, y	71 (64–78)	0	66 (58–71)	77 (72–81)	< 0.001
Female sex	25%	0	21%	29%	< 0.001
Body mass index, kg/m^2^	24 (22–26)	0	23 (21–25)	25 (23–28)	< 0.001
Acute coronary syndrome	44%	0	47%	40%	< 0.001
Comorbidities					
Hypertension	83%	2 (0.1)	79%	86%	< 0.001
Diabetes mellitus	42%	18 (0.5)	41%	44%	0.050
Dyslipidemia	79%	3 (0.1)	78%	81%	0.052
Atrial fibrillation	7%	0	0%	16%	< 0.001
Chronic kidney disease	40%	1 (0.0)	28%	54%	< 0.001
Hemodialysis	4%	1 (0.0)	5%	4%	0.046
Stroke	9%	5 (0.2)	7%	12%	< 0.001
Anemia	49%	237 (7.2)	43%	56%	< 0.001
Peripheral arterial disease	7%	8 (0.2)	7%	7%	0.776
Cancer	10%	4 (0.1)	9%	12%	0.003
Current tobacco use	21%	29 (0.9)	27%	14%	< 0.001
Previous myocardial infarction	12%	4 (0.1)	12%	11%	0.573
Past PCI	15%	3 (0.1)	12%	18%	< 0.001
Past CABG	3%	4 (0.1)	2%	4%	0.029
Systolic blood pressure, mmHg	130 (117–145)	46 (1.5)	130 (116–146)	131 (118–144)	0.496
Diastolic blood pressure, mmHg	72 (63–81)	48 (1.5)	74 (64–84)	70 (62–79)	< 0.001
Heart rate, beats/min	70 (62–80)	56 (1.7)	71 (63–81)	70 (61–81)	0.003
Echocardiographic data					
LAD, mm	38 (33–42)	2049 (62)	36 (32–40)	40 (35–44)	< 0.001
LVEDD, mm	47 (43–51)	0	47 (43–50)	47 (43–51)	0.101
LVEF, %	65 (60–71)	0	65 (60–71)	65 (60–71)	0.686
LVMI, g/m^2^	101 (85–121)	13 (0.4)	98 (83–117)	106 (89–125)	< 0.001
IVSd, mm	10 (9–11)	0	10 (9–11)	11 (10–12)	< 0.001
PWd, mm	10 (9–11)	13 (0.4)	10 (9–11)	10 (9–12)	< 0.001
Laboratory data					
White blood cell, ×10^3^/µL	6.9 (5.8–8.4)	238 (7.2)	7.1 (6.0–8.7)	6.7 (5.6–8.0)	< 0.001
Hemoglobin, g/dL	13 (11–14)	237 (7.2)	13 (12–14)	12 (11–14)	< 0.001
HbA1c, %	6.1 (5.7–6.9)	231 (7.0)	6.0 (5.7–6.8)	6.2 (5.8–6.9)	< 0.001
Albumin, g/dL	3.9 (3.5–4.2)	249 (7.5)	3.9 (3.6–4.2)	3.8 (3.5–4.1)	< 0.001
Total cholesterol, mg/dL	171 (148–196)	77 (2.3)	174 (151–201)	167 (146–191)	< 0.001
LDL cholesterol, mg/dL	96 (77–117)	33 (1.0)	97 (79–120)	93 (76–114)	< 0.001
HDL cholesterol, mg/dL	46 (39–55)	56 (1.7)	46 (39–56)	45 (38–54)	0.002
Triglyceride, mg/dL	120 (87–169)	27 (0.8)	122 (87–175)	119 (87–163)	0.105
Creatinine, mg/dL	0.9 (0.7–1.1)	1 (0.0)	0.8 (0.7–1.0)	0.9 (0.8–1.1)	< 0.001
BUN, mg/dL	16 (13–20)	238 (7.2)	15 (13–19)	17 (14–21)	< 0.001
eGFR, mL/min/1.73m^2^	65 (51–78)	1 (0.0)	71 (58–83)	58 (47–70)	< 0.001
BNP, pg/mL	53 (23–134)	496 (15.0)	40 (17–102)	69 (33–168)	< 0.001
CRP, mg/dL	0.5 (0.1–2.1)	266 (8.0)	0.5 (0.1–2.1)	0.4 (0.1–2.1)	0.770
Medications					
Statin	86%	0	85%	87%	0.323
β-blocker	63%	0	65%	62%	0.194
ACE inhibitor/ARB	68%	0	67%	69%	0.122
SGLT2 inhibitor	2%	2 (0.1)	3%	2%	0.105
MRA	7%	2 (0.1)	6%	8%	0.109
Diuretic	18%	0	14%	23%	< 0.001
Long-acting nitrate	16%	2 (0.1)	15%	17%	0.221

Values are presented as median (interquartile range) or %. ACE indicates angiotensin-converting enzyme; ARB, angiotensin II type 1 receptor blocker; BNP, B-type natriuretic peptide; BUN, blood urea nitrogen; CABG, coronary artery bypass graft; CRP, C-reactive protein; eGFR, estimated glomerular filtration rate; HbA1c, glycated hemoglobin; HDL, high-density lipoprotein; IVSd, interventricular wall thickness at end-diastole; LAD, left atrial dimension; LDL, low-density lipoprotein; LVEDD, left ventricular end-diastolic dimension; LVEF, left ventricular ejection fraction; LVMI, left ventricular mass index; MRA, mineralocorticoid receptor antagonist; PCI, percutaneous coronary intervention; PWd, left ventricular posterior wall thickness at end-diastole; and SGLT2, sodium-glucose cotransporter type 2

Compared with the analyzed cohort, patients with LVEF < 50% had more AF, renal dysfunction, anemia, previous myocardial infarction, larger LV dimensions, and higher BNP levels, whereas those with unavailable LVEF were slightly older and had more chronic kidney disease and prior coronary revascularization (*Supplementary Table 1*).

### Correlation between the HFpEF-ABA score and BNP or LAD

**Fig. 2:**
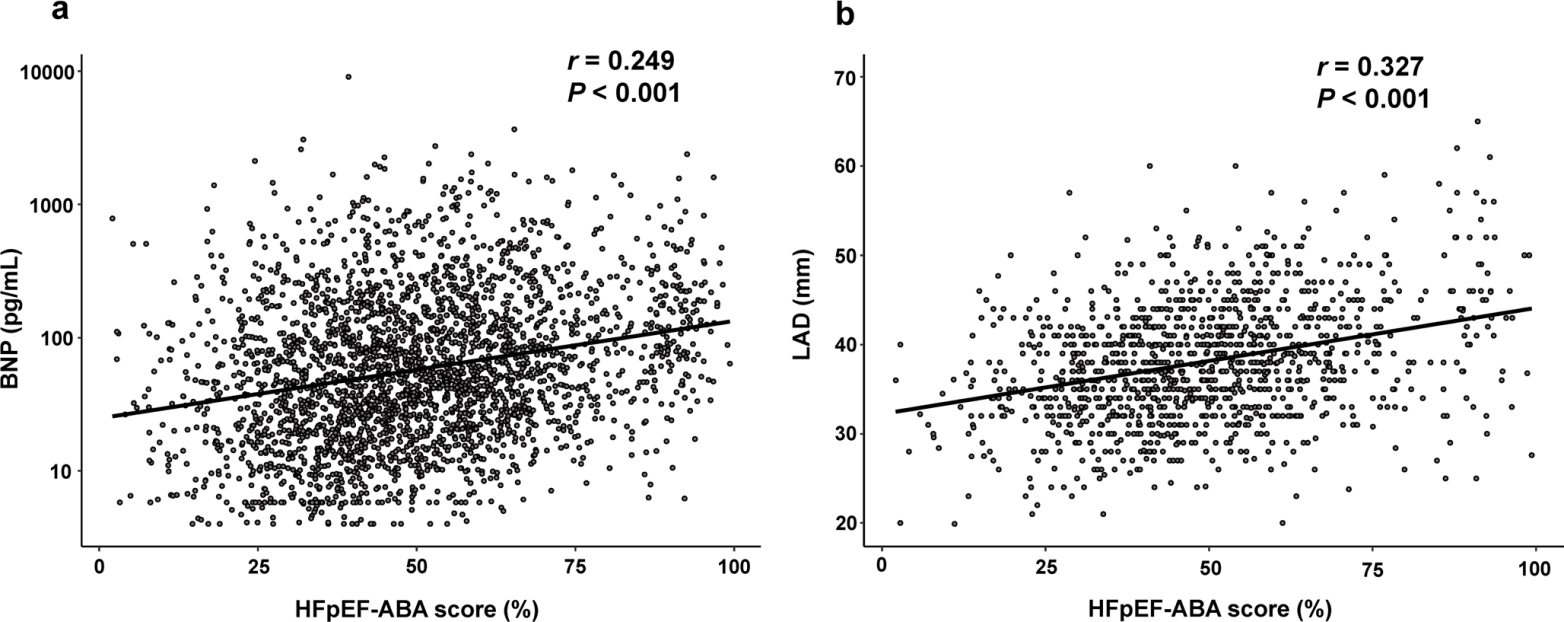
Correlation of the HFpEF-ABA score with BNP levels and LAD. Correlations between the HFpEF-ABA score and BNP levels (**a**) and LAD (**b**). BNP, B-type natriuretic peptide; LAD, left atrial dimension

Correlations between the HFpEF-ABA score and BNP level or LAD are shown in Fig. 2. The HFpEF-ABA score was significantly and positively correlated with both BNP level and LAD.

### Prognostic analysis

During a median follow-up of 721 days, 275 patients experienced the primary endpoint —119 in the low HFpEF-ABA score group and 156 in the high HFpEF-ABA score group. Multivariable Cox analysis showed that the HFpEF-ABA score was significantly associated with the primary endpoint, regardless of whether it was treated as a continuous or categorical variable (Table 2). In subgroup analyses stratified by clinical presentation (ACS and CCS), the association between the HFpEF-ABA score and the primary endpoint did not significantly differ according to clinical presentation (*Supplementary Table 2*). In a sensitivity analysis restricted to variables with *P* < 0.05 in univariable analysis, the association of the HFpEF-ABA score with the primary endpoint was attenuated: the continuous score was no longer statistically significant, whereas the ≥ 50% category remained marginally significant (*Supplementary Table 3*). In another sensitivity analysis using a 75% probability threshold, the higher-score category remained significantly associated with the primary endpoint (*Supplementary Table 4*). In a forced-in 8-variable baseline clinical model, addition of the HFpEF-ABA score yielded only a minimal increase in Harrell’s C, although the likelihood ratio test suggested a modest improvement in model fit (*Supplementary Table 5*).

**Fig. 3:**
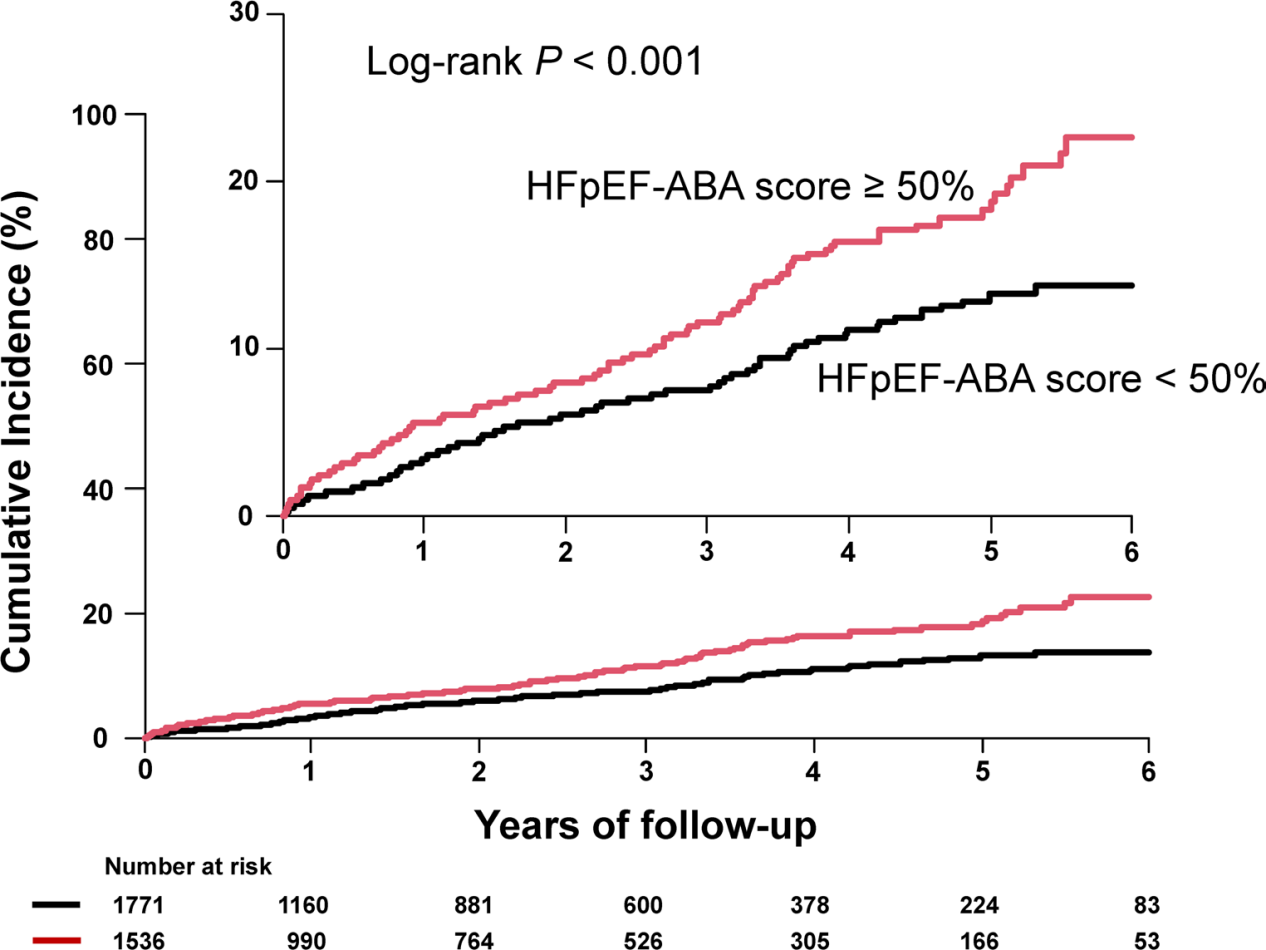
Time-to-event curves for the primary endpoint. Kaplan–Meier curves for the primary endpoint, stratified by the HFpEF-ABA score

**Fig. 4:**
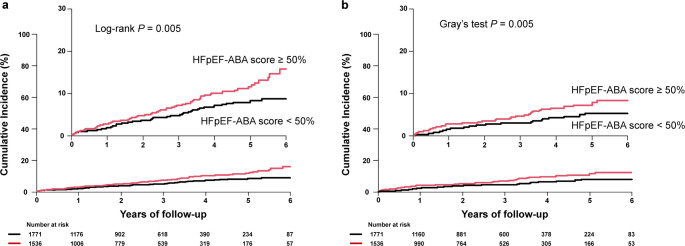
Time-to-event curves for the secondary endpoints. Kaplan–Meier curves for all-cause death (**a**) and cumulative incidence curves for unplanned hospitalization for worsening heart failure accounting for the competing risk of death (**b**), stratified by the HFpEF-ABA score

As shown in Fig. 3, the risk of the primary endpoint was significantly higher in patients with a high HFpEF-ABA score than in those with a low score. Furthermore, when the individual components of the primary endpoint were evaluated separately, patients with a high HFpEF-ABA score showed a higher incidence of all-cause death and unplanned hospitalization for HF (Fig. 4). In an exploratory Cox analysis restricted to the low HFpEF-ABA score group, male sex, diabetes mellitus, peripheral arterial disease, cancer, lower hemoglobin, and higher BNP levels were independently associated with the primary endpoint (*Supplementary Table 6*).

**Table 2:** Cox Proportional Hazard Analysis for the Primary Endpoint

	Univariable analysis		Multivariable analysis			
			Model 1		Model 2	
	HR (95% CI)	*P* value	HR (95% CI)	*P* value	HR (95% CI)	*P* value
Female sex (reference: male)	1.01 (0.77–1.34)	0.920	0.61 (0.44–0.85)	0.003	0.60 (0.44–0.83)	0.003
Acute coronary syndrome (reference: none)	0.85 (0.67–1.07)	0.174	1.02 (0.78–1.35)	0.862	1.03 (0.78–1.35)	0.856
Hypertension (reference: none)	0.98 (0.71–1.34)	0.883	0.72 (0.51–1.02)	0.069	0.73 (0.52–1.03)	0.076
Diabetes mellitus (reference: none)	1.63 (1.29–2.07)	< 0.001	1.33 (1.04–1.72)	0.026	1.31 (1.02–1.69)	0.035
Dyslipidemia (reference: none)	0.70 (0.53–0.91)	0.009	1.05 (0.78–1.41)	0.763	1.04 (0.77–1.40)	0.804
Stroke (reference: none)	1.26 (0.87–1.83)	0.223	0.78 (0.53–1.15)	0.208	0.77 (0.52–1.13)	0.184
Peripheral arterial disease (reference: none)	2.70 (1.97–3.70)	< 0.001	1.63 (1.16–2.29)	0.006	1.68 (1.20–2.37)	0.003
Cancer (reference: none)	1.93 (1.43–2.61)	< 0.001	1.85 (1.34–2.56)	< 0.001	1.84 (1.33–2.55)	< 0.001
Current tobacco use (reference: none)	0.84 (0.62–1.15)	0.280	0.96 (0.69–1.33)	0.811	0.97 (0.70–1.35)	0.870
Previous myocardial infarction (reference: none)	0.81 (0.55–1.20)	0.296	0.86 (0.57–1.28)	0.453	0.86 (0.57–1.28)	0.456
Heart rate (per 1 beat/min increase)	1.01 (1.00–1.02)	0.007	1.01 (1.00–1.01)	0.198	1.01 (1.00–1.01)	0.166
LVEDD (per 1 mm increase)	1.01 (0.99–1.03)	0.196	0.97 (0.95–1.00)	0.023	0.97 (0.95–0.99)	0.019
LVEF (per 1% increase)	0.98 (0.96–0.99)	0.002	1.00 (0.98–1.01)	0.711	1.00 (0.98–1.01)	0.673
LVMI (per 1 g/m^2^ increase)	1.01 (1.01–1.01)	< 0.001	1.00 (1.00–1.01)	0.032	1.01 (1.00–1.01)	0.025
White blood cell (per 1 × 10^3^/µL increase)	1.05 (1.00–1.11)	0.050	1.07 (1.02–1.13)	0.009	1.07 (1.01–1.12)	0.013
Hemoglobin (per 1 g/dL increase)	0.63 (0.59–0.69)	< 0.001	0.76 (0.69–0.83)	< 0.001	0.76 (0.69–0.83)	< 0.001
BUN (per 10-fold mg/dL increase)	35.14 (19.89–62.09)	< 0.001	5.84 (1.75–19.46)	0.005	5.55 (1.68–18.35)	0.006
eGFR (per 1 mL/min/1.73m^2^ increase)	0.98 (0.97–0.98)	< 0.001	1.01 (1.00–1.01)	0.221	1.01 (1.00–1.01)	0.230
BNP (per 10-fold pg/mL increase)	4.01 (3.24–4.95)	< 0.001	2.33 (1.74–3.11)	< 0.001	2.33 (1.75–3.11)	< 0.001
HFpEF-ABA score probability (per 10% increase)	1.12 (1.05–1.19)	< 0.001	1.07 (1.00–1.14)	0.043	―	
HFpEF-ABA score probability ≥ 50% (reference: <50%)	1.56 (1.23–1.98)	< 0.001	―		1.37 (1.07–1.76)	0.015

BNP indicates B-type natriuretic peptide; BUN, blood urea nitrogen; CI, confidence interval; eGFR, estimated glomerular filtration rate; HR, hazard ratio; LVEDD, left ventricular end-diastolic dimension; LVEF, left ventricular ejection fraction; and LVMI, left ventricular mass index

Further analyses showed that, among single-marker models, BNP had the highest Harrell’s C for the primary endpoint, followed by LVMI and the HFpEF-ABA score. Adding the HFpEF-ABA score to BNP did not improve Harrell’s C, whereas adding it to LVMI yielded only a modest increase (*Supplementary Table 7*). At the component level, AF remained significantly associated with the primary endpoint in a Cox model simultaneously including age, BMI, and AF, but AF was not the strongest individual component based on Wald chi-square statistics. Consistently, the HFpEF-ABA score showed only a numerically higher Harrell’s C than AF alone, without a statistically significant difference (*Supplementary Tables 8 and 9*).

## Discussion

HF is one of the major determinants of prognosis in patients with CAD. A considerable proportion of CAD patients who later develop HF have been reported to have HFpEF. Furthermore, there has even been a report demonstrating a temporal trend toward an increasing prevalence of HFpEF compared with HF with reduced ejection fraction in patients with CAD [20, 21]. Therefore, screening for the presence of preclinical or early-stage HFpEF in patients with CAD is of paramount importance for improving prognosis. In this study, we showed that the HFpEF-ABA score, which can be calculated from three easily obtainable variables, is useful for identifying CAD patients with subclinical cardiac abnormalities consistent with HFpEF. In addition, this score was also useful for predicting poor clinical outcomes. To the best of our knowledge, this is the first study to demonstrate the clinical utility of the HFpEF-ABA score for patient phenotyping and risk stratification specifically in patients with CAD.

The HFpEF-ABA score was created as a simple diagnostic score to estimate the probability of HFpEF that can be calculated without imaging data, thereby facilitating broad clinical applicability. Although all patients enrolled in the present study underwent echocardiographic evaluation as part of the inclusion criteria, real-world practice is characterized by substantial variability in the use of echocardiography among patients with CAD [8], underscoring the clinical relevance of this score. Although the HFpEF-ABA score does not include data on echocardiography or natriuretic peptide levels, it has been shown to provide diagnostic performance comparable to that of established HFpEF scores such as H_2_FPEF and HFA-PEFF scores [9]. Moreover, prior evidence has suggested that the HFpEF-ABA score is useful for identifying patients with underlying preclinical or early-stage HFpEF even among those without definitive HFpEF [22]. Recently, Reddy et al. demonstrated that the HFpEF-ABA score can identify a subset of patients with group 1 pulmonary hypertension who exhibit HFpEF-like physiological characteristics and a higher risk of adverse outcomes [23]. Our results expand on their report by showing that the HFpEF-ABA score is also useful for identifying patients with HFpEF-related phenotypic abnormalities and predicting poor clinical outcomes, even in patients with CAD.

Modern management of ACS, including urgent coronary angiography and primary PCI, has resulted in a substantial proportion of patients presenting with preserved or only mildly reduced LV systolic function [24]. Recent registry data have also shown that the predominant subtypes of HF after ACS are HF with non-reduced ejection fraction [25]. However, only a few studies have evaluated risk models predicting HF hospitalization specifically in ACS patients with preserved LVEF; in the study by Cordero et al., older age and AF were independent predictors of HF hospitalization, although BMI data were not available in their study [26]. In contrast, several CCS studies have examined risk models in cohorts including a substantial proportion of patients with preserved LVEF at baseline. Lewis et al. reported that older age and higher BMI were independent predictors of HF development [27], whereas Lamblin et al. demonstrated that older age, higher BMI, and AF were associated with an increased risk of HF hospitalization [3]. Parma et al. further showed that older age and AF were independent predictors of HF-related outcomes, and that BMI demonstrated a non-linear association, with both low BMI < 20 kg/m^2^and BMI ≥ 30 kg/m² associated with worse prognosis [28]. In the present study, subgroup analyses stratified by clinical presentation showed no significant interaction between the HFpEF-ABA score and clinical presentation for the primary endpoint. Taken together with prior reports demonstrating the prognostic relevance of age, BMI, and AF in ACS or CCS, our findings suggest that the HFpEF-ABA score may serve as a simple integrative marker of HF-related risk across different clinical presentations of CAD, although the subgroup-specific estimates should be interpreted cautiously.

Although the risk of the primary endpoint was significantly higher in patients with a high HFpEF-ABA score than in those with a low score, a non-negligible number of patients in the low-score group still experienced the primary endpoint. The exploratory Cox analysis restricted to patients with low HFpEF-ABA scores suggests that residual risk in this subgroup may reflect a broader burden of cardiovascular and systemic comorbidities that is not fully captured by the HFpEF-ABA score alone. Therefore, a low HFpEF-ABA score should not be interpreted as indicating low overall clinical risk after PCI. Because the 50% threshold was selected only for descriptive and graphical stratification, we additionally examined an alternative threshold of 75%, which has been used in recent studies, and confirmed consistent results; however, these thresholds should be regarded as supportive rather than a definitive cutoff for patients with CAD. Although the HFpEF-ABA score remained independently associated with the primary endpoint after adjustment for LVMI and BNP in the primary multivariable model, discrimination analyses showed only a modest gain when it was added to LVMI and little incremental gain when it was combined with BNP. Similarly, although addition of the HFpEF-ABA score improved overall model fit, the gain in discrimination beyond the forced-in 8-variable baseline clinical model was small. Taken together, these findings suggest that the HFpEF-ABA score is best viewed as a simple and broadly applicable adjunct to clinical risk assessment rather than a substitute for natriuretic peptide measurement, echocardiographic structural assessment, or a strong incremental discriminator.

According to the AHA/ACC/HFSA guideline for the management of HF, patients with preclinical HF, defined as stage B HF, should undergo strict management of major comorbidities, including hypertension, diabetes, dyslipidemia, chronic kidney disease, and obesity [29]. Furthermore, in the latest JCS/JHFS guideline for HF, the recommendations for risk-factor management are more clearly stated, particularly highlighting the role of sodium-glucose cotransporter 2 inhibitors and the nonsteroidal mineralocorticoid receptor antagonist finerenone in patients with diabetes and chronic kidney disease to prevent HF [30]. In our cohort, patients with a high HFpEF-ABA score more frequently exhibited HFpEF-related structural and biomarker abnormalities and had worse clinical outcomes. In this context, the HFpEF-ABA score may serve as a simple screening tool in the post-PCI setting to identify patients who may warrant closer follow-up, more careful evaluation for preclinical or early-stage HFpEF when clinically indicated, and more intensive implementation of these guideline-recommended preventive strategies. Integrating the HFpEF-ABA score into electronic health records may further facilitate the real-time recognition of such high-risk patients and support more tailored post-PCI management in daily practice. Whether HFpEF-ABA score–guided risk stratification and subsequent management can prevent progression to overt HFpEF and improve long-term outcomes remains to be determined and requires further prospective investigation.

This study has several limitations. First, although we collected data on consecutive patients from seven hospitals to minimize selection bias during the study period, the retrospective design may still have introduced selection bias. In addition, reliance on existing medical records and the exclusion of patients with missing follow-up data or a missing baseline HFpEF-ABA score may have introduced additional bias and limited the validity of our findings. Furthermore, because a substantial number of patients had unavailable baseline LVEF data and were therefore excluded, selection bias related to LVEF availability cannot be ruled out, and the present findings may be most applicable to CAD patients with documented preserved LVEF rather than to the overall unselected PCI population. Second, because this study utilized a multicenter East-Asian registry, potential ethnic differences should be considered when generalizing these findings to non-Japanese populations. The HFpEF-ABA score was developed and validated mainly in predominantly White cohorts without Asian representation; although discrimination was similar in small non-White and White subgroups [9], ethnic differences in BMI distribution may affect transportability. Moreover, although participating sites included representative hospitals across multiple regions of Japan, our results may not be fully generalizable to patients treated outside these hospitals. Therefore, external validation in independent cohorts is required to confirm the robustness and generalizability of the HFpEF-ABA score across various demographics, geographic regions, and healthcare systems. Third, because we did not capture the occurrence of HF during hospitalization for the index PCI, some patients may have had unrecognized HF at baseline, which could have affected our results. Finally, we could not calculate the Global Registry of Acute Coronary Events (GRACE) risk score in patients with ACS because several required variables were unavailable. Therefore, we could not directly compare the performance of the HFpEF-ABA score with that of the GRACE score in the ACS subgroup, despite a previous report suggesting that the GRACE score can predict new-onset HF after discharge even in ACS patients without prior HF or LV dysfunction [26].

In conclusion, this study demonstrated that the HFpEF-ABA score can help identify patients with HFpEF-related phenotypic features among those with CAD and can predict adverse clinical outcomes. Further studies are warranted to validate our findings and to test whether incorporating the HFpEF-ABA score into clinical decision-making improves outcomes in patients with CAD.

## Electronic Supplementary Material

Below is the link to the electronic supplementary material.

Supplementary Material 1
