# Supplementary material for: Prognostic value of pretest probability of heart failure with preserved ejection fraction in patients with coronary artery disease: an insight from the CLIDAS-PCI database
Source: Cardiovasc Interv Ther. 2026 May 16;41(3):639–50. doi: 10.1007/s12928-026-01286-y (PMC13279665; doi:10.1007/s12928-026-01286-y)
Supplement: Supplementary file 1 — Supplementary Material 1 [file 12928_2026_1286_MOESM1_ESM.docx]

| Supplementary Table 1. Baseline Characteristics | | | | | | |
| --- | --- | --- | --- | --- | --- | --- |
|  |  |  | LVEF | | |  |
|  | Total | Missing | LVEF ≥50% | LVEF <50% | LVEF unavailable |  |
| Characteristics | (n=9314) | n (%) | (n=3307) | (n=1071) | (n=4936) | *P* value |
| Age, y | 71 (64–78) | 0 | 71 (64–78) | 70 (62–78) | 72 (65–78) | <0.001 |
| Female sex | 22% | 0 | 25% | 18% | 21% | <0.001 |
| Body mass index, kg/m^2^ | 24 (22–26) | 0 | 24 (22–26) | 23 (21–26) | 24 (22–26) | <0.001 |
| Acute coronary syndrome | 42% | 0 | 44% | 48% | 40% | <0.001 |
| Comorbidities |  |  |  |  |  |  |
| Hypertension | 82% | 21 (0.2) | 83% | 80% | 82% | 0.171 |
| Diabetes mellitus | 43% | 47 (0.5) | 42% | 47% | 43% | 0.049 |
| Dyslipidemia | 78% | 20 (0.2) | 79% | 74% | 79% | 0.003 |
| Atrial fibrillation | 9% | 0 | 7% | 12% | 10% | <0.001 |
| Chronic kidney disease | 46% | 21 (0.2) | 40% | 58% | 47% | <0.001 |
| Hemodialysis | 7% | 14 (0.2) | 4% | 11% | 7% | <0.001 |
| Stroke | 11% | 16 (0.2) | 9% | 11% | 12% | 0.001 |
| Anemia | 51% | 523 (5.6) | 49% | 61% | 51% | <0.001 |
| Peripheral arterial disease | 8% | 876 (9.4) | 7% | 7% | 10% | <0.001 |
| Cancer | 10% | 15 (0.2) | 10% | 11% | 10% | 0.674 |
| Current tobacco use | 20% | 75 (0.8) | 21% | 26% | 19% | <0.001 |
| Previous myocardial infarction | 15% | 16 (0.2) | 12% | 23% | 16% | <0.001 |
| Past PCI | 21% | 11 (0.1) | 15% | 17% | 26% | <0.001 |
| Past CABG | 5% | 7 (0.1) | 3% | 7% | 7% | <0.001 |
| Systolic blood pressure, mmHg | 128 (115–143) | 184 (2.0) | 130 (117–145) | 124 (110–143) | 127 (114–141) | <0.001 |
| Diastolic blood pressure, mmHg | 70 (62–81) | 190 (2.0) | 72 (63–81) | 71 (62–83) | 70 (61–80) | <0.001 |
| Heart rate, beats/min | 71 (62–81) | 227 (2.4) | 70 (62–80) | 78 (67–90) | 70 (62–80) | <0.001 |
| Echocardiographic data |  |  |  |  |  |  |
| LAD, mm | 39 (34–43) | 7544 (81.0) | 38 (33–42) | 41 (36–46) | 38 (35–40) | <0.001 |
| LVEDD, mm | 48 (44–53) | 4905 (52.7) | 47 (43–51) | 55 (49–61) | 49 (46–51) | <0.001 |
| LVEF, % | 62 (50–69) | 4936 (53.0) | 65 (60–71) | 40 (33–46) | ― | <0.001 |
| LVMI, g/m^2^ | 106 (88–129) | 4924 (52.9) | 101 (85–121) | 126 (104–150) | 99 (86–122) | <0.001 |
| IVSd, mm | 10 (9–11) | 4864 (52.2) | 10 (9–11) | 10 (9–11) | 10 (9–11) | <0.001 |
| PWd, mm | 10 (9–11) | 4879 (52.4) | 10 (9–11) | 10 (9–11) | 10 (9–10) | <0.001 |
| Laboratory data |  |  |  |  |  |  |
| White blood cell, ×10^3^/μL | 6.9 (5.8–8.3) | 526 (5.6) | 6.9 (5.8–8.4) | 7.4 (6.2–9.1) | 6.9 (5.7–8.2) | <0.001 |
| Hemoglobin, g/dL | 13 (11–14) | 523 (5.6) | 13 (11–14) | 12 (11–14) | 13 (11–14) | <0.001 |
| HbA1c, % | 6.1 (5.7–6.8) | 767 (8.2) | 6.1 (5.7–6.9) | 6.2 (5.8–7.0) | 6.1 (5.7–6.7) | <0.001 |
| Albumin, g/dL | 3.9 (3.5–4.2) | 563 (6.0) | 3.9 (3.5–4.2) | 3.5 (3.2–3.9) | 3.9 (3.6–4.2) | <0.001 |
| Total cholesterol, mg/dL | 166 (145–190) | 259 (2.8) | 171 (148–196) | 162 (139–185) | 164 (144–186) | <0.001 |
| LDL cholesterol, mg/dL | 93 (75–113) | 148 (1.6) | 96 (77–117) | 93 (74–113) | 91 (74–109) | <0.001 |
| HDL cholesterol, mg/dL | 45 (38–54) | 216 (2.3) | 46 (39–55) | 42 (35–51) | 45 (38–54) | <0.001 |
| Triglyceride, mg/dL | 117 (86–165) | 126 (1.4) | 120 (87–169) | 105 (79–146) | 119 (87–166) | <0.001 |
| Creatinine, mg/dL | 0.9 (0.7–1.1) | 21 (0.2) | 0.9 (0.7–1.1) | 1.0 (0.8–1.5) | 0.9 (0.8–1.1) | <0.001 |
| BUN, mg/dL | 17 (14–21) | 526 (5.6) | 16 (13–20) | 20 (15–29) | 17 (14–21) | <0.001 |
| eGFR, mL/min/1.73m^2^ | 62 (48–75) | 21 (0.2) | 65 (51–78) | 56 (35–70) | 61 (47–73) | <0.001 |
| BNP, pg/mL | 71 (28–207) | 1114 (12.0) | 53 (23–134) | 301 (110–722) | 67 (28–187) | <0.001 |
| CRP, mg/dL | 0.5 (0.1–2.0) | 573 (6.2) | 0.5 (0.1–2.1) | 1.3 (0.3–3.8) | 0.4 (0.1–1.5) | <0.001 |
| Medications |  |  |  |  |  |  |
| Statin | 82% | 0 | 86% | 83% | 78% | <0.001 |
| β-blocker | 61% | 0 | 63% | 78% | 56% | <0.001 |
| ACE inhibitor/ARB | 66% | 0 | 68% | 77% | 62% | <0.001 |
| SGLT2 inhibitor | 2% | 35 (0.4) | 2% | 3% | 1% | <0.001 |
| MRA | 10% | 35 (0.4) | 7% | 29% | 8% | <0.001 |
| Diuretic | 23% | 0 | 18% | 51% | 21% | <0.001 |
| Long-acting nitrate | 15% | 35 (0.4) | 16% | 12% | 14% | 0.007 |
| Values are presented as median (interquartile range) or %. ACE indicates angiotensin-converting enzyme; ARB, angiotensin II type 1 receptor blocker; BNP, B-type natriuretic peptide; BUN, blood urea nitrogen; CABG, coronary artery bypass graft; CRP, C-reactive protein; eGFR, estimated glomerular filtration rate; HbA1c, glycated hemoglobin; HDL, high-density lipoprotein; IVSd, interventricular wall thickness at end-diastole; LAD, left atrial dimension; LDL, low-density lipoprotein; LVEDD, left ventricular end-diastolic dimension; LVEF, left ventricular ejection fraction; LVMI, left ventricular mass index; MRA, mineralocorticoid receptor antagonist; PCI, percutaneous coronary intervention; PWd, left ventricular posterior wall thickness at end-diastole; and SGLT2, sodium-glucose cotransporter type 2. | | | | | | |

| Supplementary Table 2. Cox Proportional Hazard Analysis for the Primary Endpoint | | | | |  |
| --- | --- | --- | --- | --- | --- |
|  | Multivariable analysis | | |  |  |
|  | ACS |  | CCS |  |  |
|  | HR (95% CI) |  | HR (95% CI) | *P* for interaction |  |
| HFpEF-ABA score probability (per 10 % increase) | 1.02 (0.93–1.13) |  | 1.10 (1.01–1.21) | 0.369 |  |
| HFpEF-ABA score probability ≥50% (reference: <50%) | 1.24 (0.85–1.81) |  | 1.54 (1.09–2.19) | 0.350 |  |
| Multivariable models were adjusted for the same variables as in Table 2 except for acute coronary syndrome. *P* for interaction was obtained from a Cox model including an interaction term between HFpEF-ABA score and clinical presentation. 　ACS indicates acute coronary syndrome; CCS, chronic coronary syndrome; CI, confidence interval; and HR, hazard ratio. | | | | |  |
|  |  |  |  |  |  |

| Supplementary Table 3. Cox Proportional Hazard Analysis for the Primary Endpoint | | | | | |  |
| --- | --- | --- | --- | --- | --- | --- |
|  | Multivariable analysis | | | | |  |
|  | Model 1 | |  | Model 2 | |  |
|  | HR (95% CI) | *P* value |  | HR (95% CI) | *P* value |  |
| Diabetes mellitus (reference: none) | 1.27 (0.99–1.62) | 0.065 |  | 1.25 (0.97–1.60) | 0.082 |  |
| Dyslipidemia (reference: none) | 0.95 (0.72–1.26) | 0.732 |  | 0.95 (0.72–1.25) | 0.698 |  |
| Peripheral arterial disease (reference: none) | 1.72 (1.24–2.39) | 0.001 |  | 1.77 (1.27–2.46) | 0.001 |  |
| Cancer (reference: none) | 1.85 (1.35–2.52) | <0.001 |  | 1.84 (1.35–2.52) | <0.001 |  |
| Heart rate (per 1 beat/min increase) | 1.01 (1.00–1.01) | 0.133 |  | 1.01 (1.00–1.01) | 0.116 |  |
| LVEF (per 1 % increase) | 1.00 (0.98–1.01) | 0.620 |  | 1.00 (0.98–1.01) | 0.597 |  |
| LVMI (per 1 g/m^2^ increase) | 1.00 (1.00–1.01) | 0.307 |  | 1.00 (1.00–1.01) | 0.284 |  |
| Hemoglobin (per 1 g/dL increase) | 0.79 (0.72–0.87) | <0.001 |  | 0.79 (0.72–0.87) | <0.001 |  |
| BUN (per 10-fold mg/dL increase) | 7.64 (2.45–23.77) | 0.001 |  | 7.37 (2.38–22.78) | 0.001 |  |
| eGFR (per 1 mL/min/1.73m^2^ increase) | 1.01 (1.00–1.02) | 0.098 |  | 1.01 (1.00–1.02) | 0.105 |  |
| BNP (per 10-fold pg/mL increase) | 2.22 (1.68–2.92) | <0.001 |  | 2.21 (1.68–2.91) | <0.001 |  |
| HFpEF-ABA score probability (per 10 % increase) | 1.05 (0.99–1.12) | 0.112 |  | ― | |  |
| HFpEF-ABA score probability ≥50% (reference: <50%) | ― | |  | 1.28 (1.01–1.64) | 0.046 |  |
| Covariates in this sensitivity analysis were restricted to variables with *P*<0.05 in univariable analysis in Table 2. 　BNP indicates B-type natriuretic peptide; BUN, blood urea nitrogen; CI, confidence interval; eGFR, estimated glomerular filtration rate; HR, hazard ratio; LVEF, left ventricular ejection fraction; and LVMI, left ventricular mass index. | | | | | |  |
|  |  |  |  |  |  |  |

| Supplementary Table 4. Sensitivity Analysis of the Multivariable Cox Proportional Hazard Analysis for the Primary Endpoint Using a 75% HFpEF-ABA Probability Threshold | | |  |
| --- | --- | --- | --- |
|  | Multivariable analysis | |  |
|  | HR (95% CI) | *P* value |  |
| Female sex (reference: male) | 0.63 (0.46–0.86) | 0.004 |  |
| Acute coronary syndrome (reference: none) | 1.01 (0.77–1.32) | 0.932 |  |
| Hypertension (reference: none) | 0.73 (0.52–1.02) | 0.066 |  |
| Diabetes mellitus (reference: none) | 1.36 (1.06–1.75) | 0.018 |  |
| Dyslipidemia (reference: none) | 1.05 (0.78–1.40) | 0.763 |  |
| Stroke (reference: none) | 0.78 (0.53–1.15) | 0.213 |  |
| Peripheral arterial disease (reference: none) | 1.60 (1.14–2.25) | 0.007 |  |
| Cancer (reference: none) | 1.83 (1.33–2.51) | <0.001 |  |
| Current tobacco use (reference: none) | 0.95 (0.69–1.31) | 0.751 |  |
| Previous myocardial infarction (reference: none) | 0.83 (0.56–1.24) | 0.373 |  |
| Heart rate (per 1 beat/min increase) | 1.01 (1.00–1.01) | 0.210 |  |
| LVEDD (per 1 mm increase) | 0.97 (0.95–1.00) | 0.028 |  |
| LVEF (per 1 % increase) | 1.00 (0.98–1.01) | 0.668 |  |
| LVMI (per 1 g/m^2^ increase) | 1.01 (1.00–1.01) | 0.030 |  |
| White blood cell (per 1×10^3^/μL increase) | 1.07 (1.02–1.13) | 0.011 |  |
| Hemoglobin (per 1 g/dL increase) | 0.76 (0.70–0.84) | <0.001 |  |
| BUN (per 10-fold mg/dL increase) | 6.09 (1.84–20.21) | 0.004 |  |
| eGFR (per 1 mL/min/1.73m^2^ increase) | 1.01 (1.00–1.01) | 0.229 |  |
| BNP (per 10-fold pg/mL increase) | 2.26 (1.73–2.95) | <0.001 |  |
| HFpEF-ABA score probability ≥75% (reference: <75%) | 1.50 (1.07–2.10) | 0.019 |  |
| BNP indicates B-type natriuretic peptide; BUN, blood urea nitrogen; CI, confidence interval; eGFR, estimated glomerular filtration rate; HR, hazard ratio; LVEDD, left ventricular end-diastolic dimension; LVEF, left ventricular ejection fraction; and LVMI, left ventricular mass index. | | |  |
|  |  |  |  |

| Supplementary Table 5. Comparison of the Discriminative Performance for the Primary Endpoint | | | | | |  |
| --- | --- | --- | --- | --- | --- | --- |
| Variable | Harrell's C (95% CI) | ΔC (95% CI) | χ^2^ | Δχ^2^ | LR test *P* |  |
| Baseline clinical model | 0.760 (0.730–0.791) | Reference | 220.1 | Reference |  |  |
| Baseline clinical model + HFpEF-ABA | 0.762 (0.731–0.792) | 0.001 (-0.041–0.043) | 226.5 | 6.4 | 0.012 |  |
| Baseline clinical model included female sex, acute coronary syndrome, hypertension, diabetes mellitus, peripheral arterial disease, cancer, hemoglobin, and estimated glomerular filtration rate. χ^2^ and Δχ^2^ indicate the global chi-square and its change, respectively. 　CI indicates confidence interval; and LR, likelihood ratio. | | | | | |  |
|  |  |  |  |  |  |  |

| Supplementary Table 6. Cox Proportional Hazard Analysis for the Primary Endpoint in Patients With Low HFpEF-ABA score | | | | | |  |
| --- | --- | --- | --- | --- | --- | --- |
|  | Univariable analysis | |  | Multivariable analysis | |  |
|  | HR (95% CI) | *P* value |  | HR (95% CI) | *P* value |  |
| Female sex (reference: male) | 0.87 (0.55–1.36) | 0.533 |  | 0.54 (0.32–0.91) | 0.023 |  |
| Acute coronary syndrome (reference: none) | 0.96 (0.67–1.37) | 0.804 |  | 0.87 (0.57–1.32) | 0.515 |  |
| Hypertension (reference: none) | 1.17 (0.73–1.90) | 0.516 |  | 0.85 (0.51–1.40) | 0.518 |  |
| Diabetes mellitus (reference: none) | 2.33 (1.61–3.37) | <0.001 |  | 1.63 (1.10–2.42) | 0.017 |  |
| Dyslipidemia (reference: none) | 0.80 (0.53–1.21) | 0.296 |  | 1.30 (0.82–2.06) | 0.269 |  |
| Stroke (reference: none) | 1.26 (0.66–2.41) | 0.480 |  | 0.67 (0.34–1.33) | 0.257 |  |
| Peripheral arterial disease (reference: none) | 3.34 (2.15–5.19) | <0.001 |  | 1.86 (1.13–3.07) | 0.016 |  |
| Cancer (reference: none) | 2.80 (1.82–4.31) | <0.001 |  | 1.81 (1.12–2.93) | 0.017 |  |
| Current tobacco use (reference: none) | 0.99 (0.66–1.50) | 0.977 |  | 1.13 (0.73–1.74) | 0.590 |  |
| Previous myocardial infarction (reference: none) | 0.72 (0.40–1.32) | 0.293 |  | 0.73 (0.39–1.34) | 0.311 |  |
| Heart rate (per 1 beat/min increase) | 1.01 (1.00–1.02) | 0.249 |  | 1.00 (0.99–1.01) | 0.757 |  |
| LVEDD (per 1 mm increase) | 1.01 (0.98–1.04) | 0.455 |  | 0.99 (0.95–1.03) | 0.699 |  |
| LVEF (per 1 % increase) | 0.97 (0.94–0.99) | 0.009 |  | 0.99 (0.96–1.01) | 0.355 |  |
| LVMI (per 1 g/m^2^ increase) | 1.01 (1.00–1.01) | 0.001 |  | 1.00 (0.99–1.01) | 0.788 |  |
| White blood cell (per 1×10^3^/μL increase) | 1.02 (0.93–1.12) | 0.659 |  | 1.03 (0.95–1.13) | 0.456 |  |
| Hemoglobin (per 1 g/dL increase) | 0.58 (0.52–0.65) | <0.001 |  | 0.66 (0.57–0.75) | <0.001 |  |
| BUN (per 10-fold mg/dL increase) | 21.64 (9.28–50.44) | <0.001 |  | 5.30 (0.84–33.47) | 0.079 |  |
| eGFR (per 1 mL/min/1.73m^2^ increase) | 0.98 (0.98–0.99) | <0.001 |  | 1.01 (1.00–1.02) | 0.228 |  |
| BNP (per 10-fold pg/mL increase) | 2.96 (2.18–4.02) | <0.001 |  | 1.64 (1.02–2.63) | 0.047 |  |
| BNP indicates B-type natriuretic peptide; BUN, blood urea nitrogen; CI, confidence interval; eGFR, estimated glomerular filtration rate; HR, hazard ratio; LVEDD, left ventricular end-diastolic dimension; LVEF, left ventricular ejection fraction; and LVMI, left ventricular mass index. | | | | | |  |
|  |  |  |  |  |  |  |

| Supplementary Table 7. Comparison of the Discriminative Performance for the Primary Endpoint | | |  |
| --- | --- | --- | --- |
| Variable | Harrell's C (95% CI) | ΔC vs HFpEF-ABA score |  |
| HFpEF-ABA alone | 0.558 (0.519–0.597) | Reference |  |
| LVMI alone | 0.606 (0.566–0.646) | 0.048 (-0.008–0.104) |  |
| BNP alone | 0.746 (0.712–0.780) | 0.188 (0.136–0.240) |  |
| HFpEF-ABA + LVMI | 0.617 (0.576–0.657) | 0.058 (0.002–0.115) |  |
| HFpEF-ABA + BNP | 0.745 (0.711–0.779) | 0.187 (0.135–0.239) |  |
| BNP indicates B-type natriuretic peptide; CI, confidence interval; and LVMI, left ventricular mass index. | | |  |
|  |  |  |  |

| Supplementary Table 8. Multivariable Cox Proportional Hazard Analysis for the Primary Endpoint Including the Individual Components of the HFpEF-ABA Score | | | |  |
| --- | --- | --- | --- | --- |
|  | HR (95% CI) | *P* value | Wald χ^2^ |  |
| Age (per 1-year increase) | 1.04 (1.02–1.05) | <0.001 | 28.22 |  |
| Body mass index (per 1 kg/m^2^ increase) | 0.90 (0.87–0.94) | <0.001 | 30.74 |  |
| Atrial fibrillation | 1.78 (1.27–2.50) | 0.001 | 11.05 |  |
| Age, body mass index, and atrial fibrillation were simultaneously entered as separate covariates. 　CI indicates confidence interval; and HR, hazard ratio. | | | |  |
|  |  |  |  |  |

| Supplementary Table 9. Comparison of the Discriminative Performance for the Primary Endpoint | | |  |
| --- | --- | --- | --- |
| Variable | Harrell's C (95% CI) | ΔC vs HFpEF-ABA score |  |
| Atrial fibrillation alone | 0.533 (0.511–0.555) | -0.025 (-0.070–0.020) |  |
| HFpEF-ABA alone | 0.558 (0.519–0.597) | Reference |  |
| CI indicates confidence interval. | | |  |
|  |  |  |  |
